# Supplementary material for: Unconventional oil and gas development and ambient particle radioactivity
Source: Nat Commun. 2020 Oct 13;11:5002. doi: 10.1038/s41467-020-18226-w (PMC7553919; doi:10.1038/s41467-020-18226-w)
Supplement: Supplementary file 1 — Supplementary Information [file 41467_2020_18226_MOESM1_ESM.pdf]

Supplementary Information

Unconventional Oil and Gas Development and Ambient Particle Radioactivity

Longxiang Li[1], Annelise Blomberg[1], John Spengler[1], Brent Coull[2], Joel Schwartz[1], Petros Koutrakis[1*]

[1]Department of Environmental Health, Harvard T.H Chan School of Public Health, Boston, Massachusetts, 02114, United States

[2]Department of Biostatistics, Harvard T.H Chan School of Public Health, Boston, Massachusetts, 02114, United States

Correspondence to: petros@hsph.harvard.edu; lol087@mail.harvard.edu.

Supplementary Notes

Supplementary Note 1. Prediction of Drilling Type

State energy agencies are the primary data source of Enverus (formerly Drillinginfo). Directional drilling has different definitions among states. For example, drilled wells and horizontally drilled wells are both grouped in a single class in Colorado as directional wells, while the two are separate in New Mexico. As a result, there is a visually remarkable difference in the percentage of horizontal wells, which are considered unconventional wells, across the state line even though they share the target geological formation. As a result, it is not reliable to assume that all directionally drilled wells are unconventional wells nationally. Besides, the raw dataset from Enverus does not provide drilling type information for more than 75% of the wells, mostly drilled before 2000. However, it is also inaccurate to assume all wells without drilling type information are conventional because drilling type is not required by a state agency but reported by the operators voluntarily. Almost all wells in Alabama do not have drilling type information. To solve these problems, we need to predict the binary drilling type based on known drilling types of nearby wells and other secondary information.

We fitted a random forest model to perform this prediction. Random forest is a regression tree-based algorithm good at capturing the non-linear relationship between the primary variable and secondary variables, thus suitable for solving this binary classification problem[1]. Secondary variables incorporated in the model include: 1) drilling type of the nearest well with known drilling type information; 2) distance to the nearest conventional/unconventional well; 3) fractions of conventional and unconventional wells of the nearest 10 wells with known drilling type; 4) O&G reservoir where the well is positioned; 5) spudding/completion time; 6) drilling depth; 7) natural gas /liquid production in the first 6 months, and; 8) production declining rate of gas/liquid. After

running a grid search for optimal performance, the parameters of this model were set as follows: the number of trees was 100, the maximum depth was 15, the minimum size of node was 5. The accuracy of this model was **99.83%** for COGD wells and **93.1%** for UOGD wells. The performance difference is potentially caused by the re-fracturing process of some conventional O&G wells. As shown in **Supplementary Figure 7**, the 10 most important covariates were: number of COGD wells within 10 km, number of UOGD wells within 10 km, the drilling type of the closest well with known drilling type, the total drilling depth (vertical drilling length plus lateral drilling length), spudding date, the first production date, completion date, the date of peak million cubic feet of gas equivalent production, the date of peak natural gas production, the date of peak barrel of oil equivalent production.

We used the Distributed Random Forest (DRF) method implemented in h2o package (version 3.26.0.2) in R (version 3.4.2) to fit the models.

Supplementary Figures

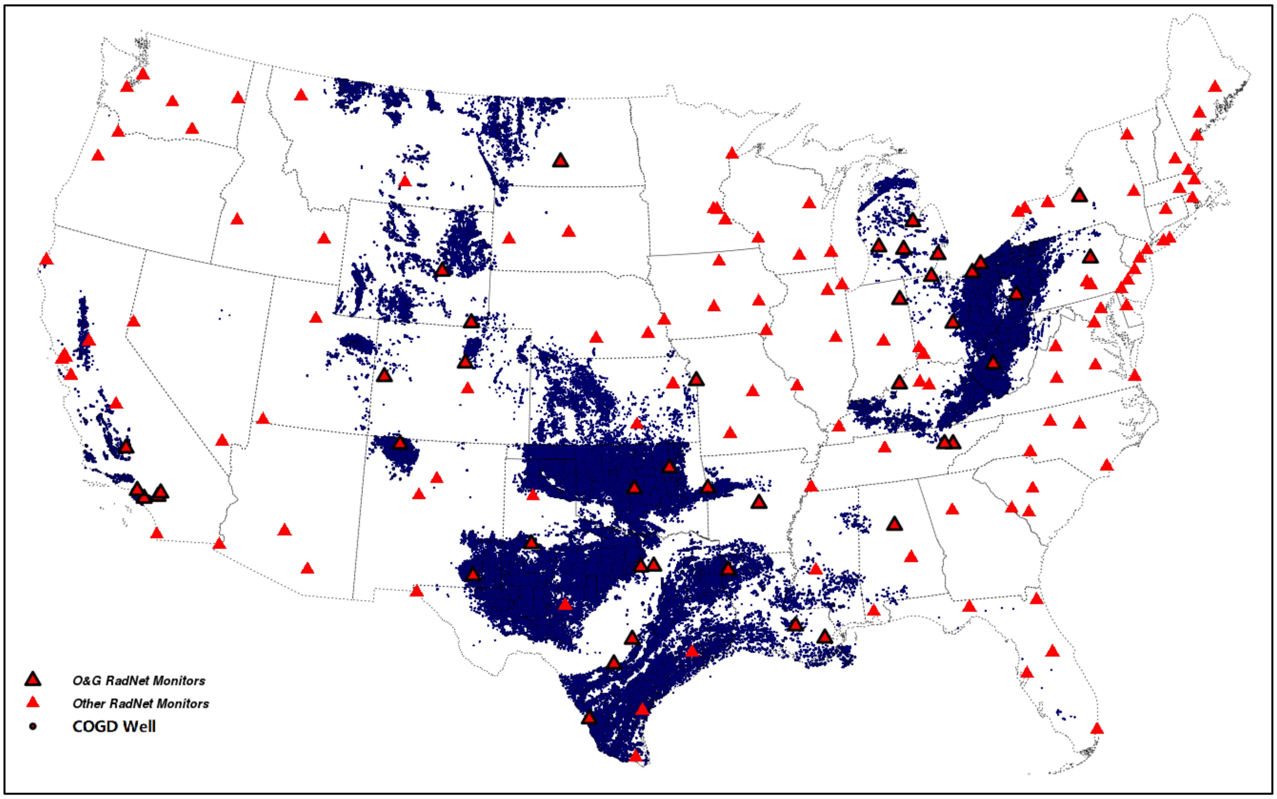

Supplementary Figure 1. The location of COGD wells completed by December 2017.

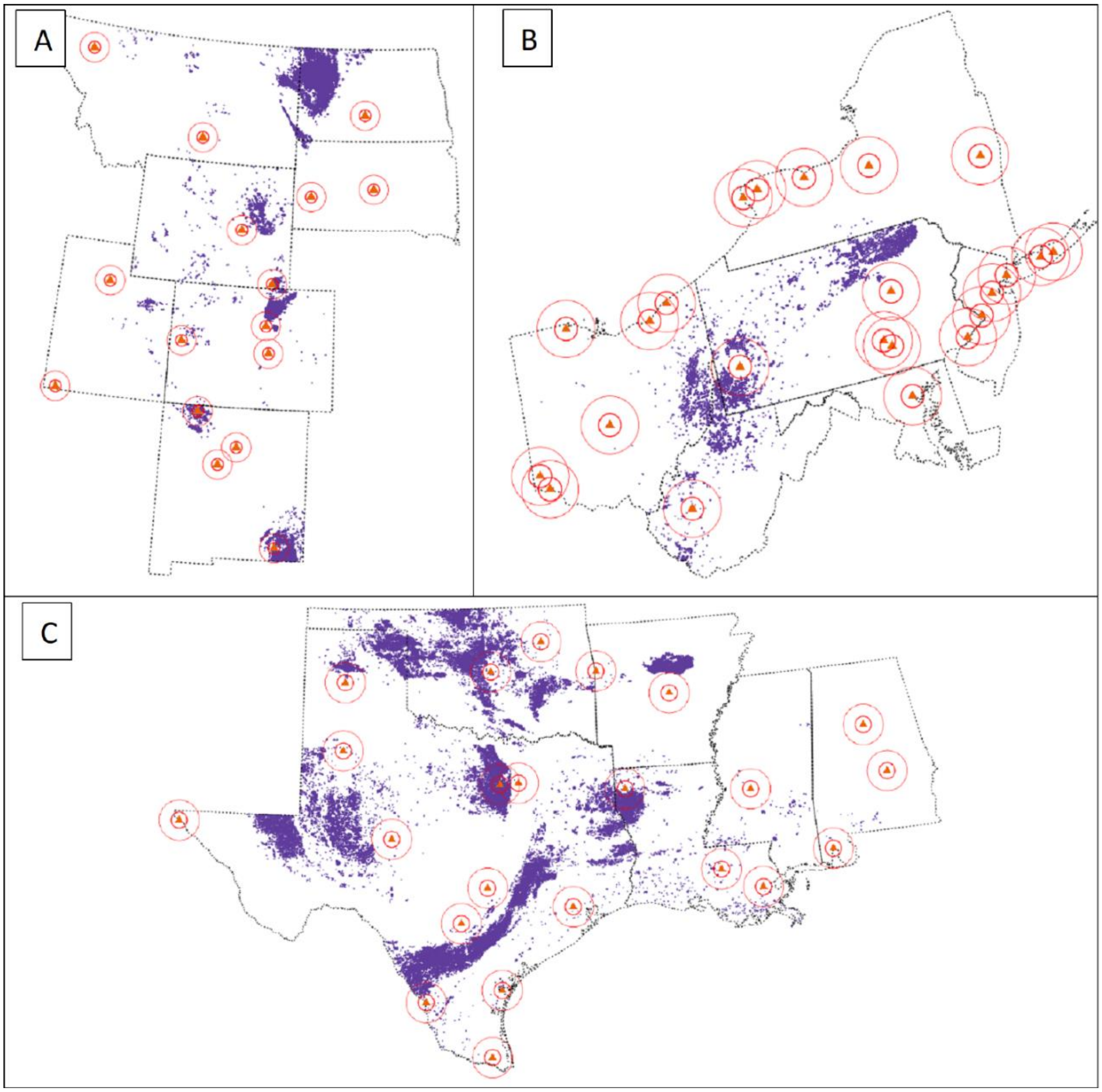

Supplementary Figure 2. Subregions of our study extent primarily determined by the shale formations.

Inner circles represent the circular buffers with a radius of 20 km. Outer circles represent the circular buffers with a radius of 50 km. Panel A shows the extent of Bakken-Niobrara subregion, which includes MT, ND, SD, WY, UT, CO, and NM; Panel B shows the extent of Marcellus-Utica subregion, which includes PA, OH, WV, NY, NJ, and MD; Panel C shows the extent of Permian-Haynesville subregion which covers TX, OK, AR, LA, MS, and AL.

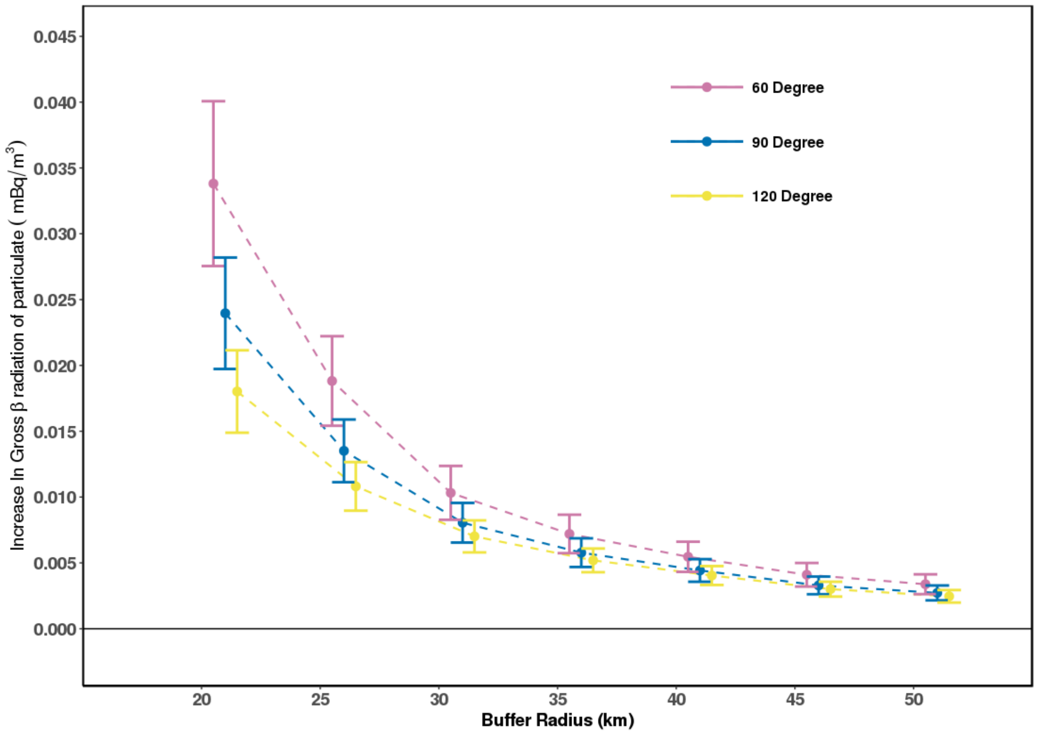

Supplementary Figure 3. The increment in PR associated with an increase of 100 upwind UOGD wells in circular sectional buffers with different central angles.

In our primary analysis, we counted the number of O&G wells within circular sectional buffers, whose central angle was 90° (As shown in **Figure 3**). We re-calculated the upwind UOGD well in buffers with two additional central angles (60° and 120°), to test the sensitivity of our estimated effects. Our results are not sensitive to the variation in angle. The estimated effects for a smaller angle (60°) were larger than our original results, while the estimated effects for a larger angle (120°) were smaller than our original results. The negative correlation between the estimated effects and buffer angle is in agreement with the atmospheric dispersion model. We also visualized the estimated effects as points and 95% CI as bars. The source data is attached in **Supplementary Table 4**.

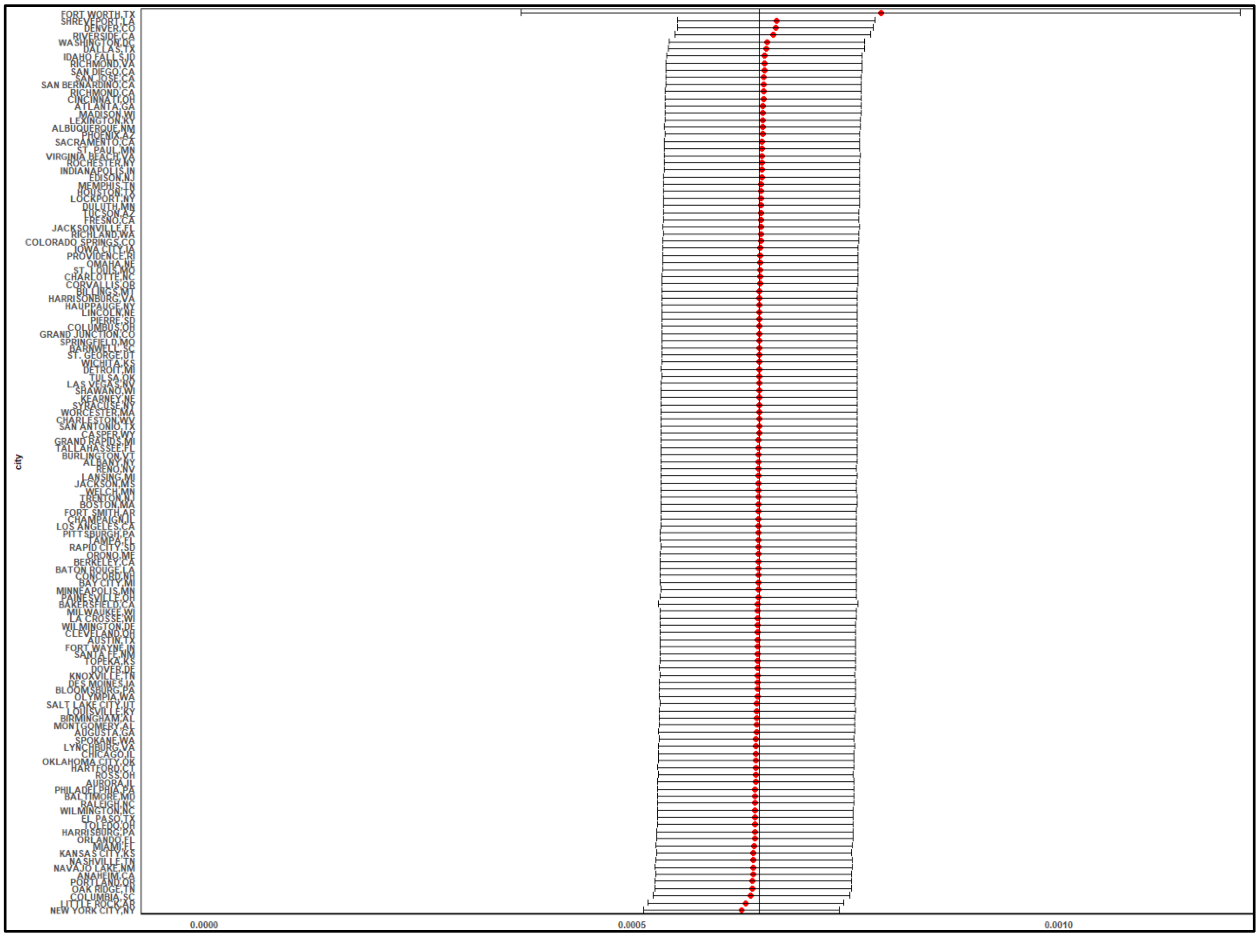

Supplementary Figure 4. The influence of omitting one monitor on the estimated association between PR and upwind UOGD well count within 20 km.

We carried out a leave-one-out analysis to investigate the likelihood that our estimated result is driven by a single RadNet monitor. Specifically, we iteratively exclude all PR measurements of a RadNet monitor and re-estimate the estimated effect with the remaining monitors using the same model formation. Our result was not sensitive to omitting a single RadNet monitor.

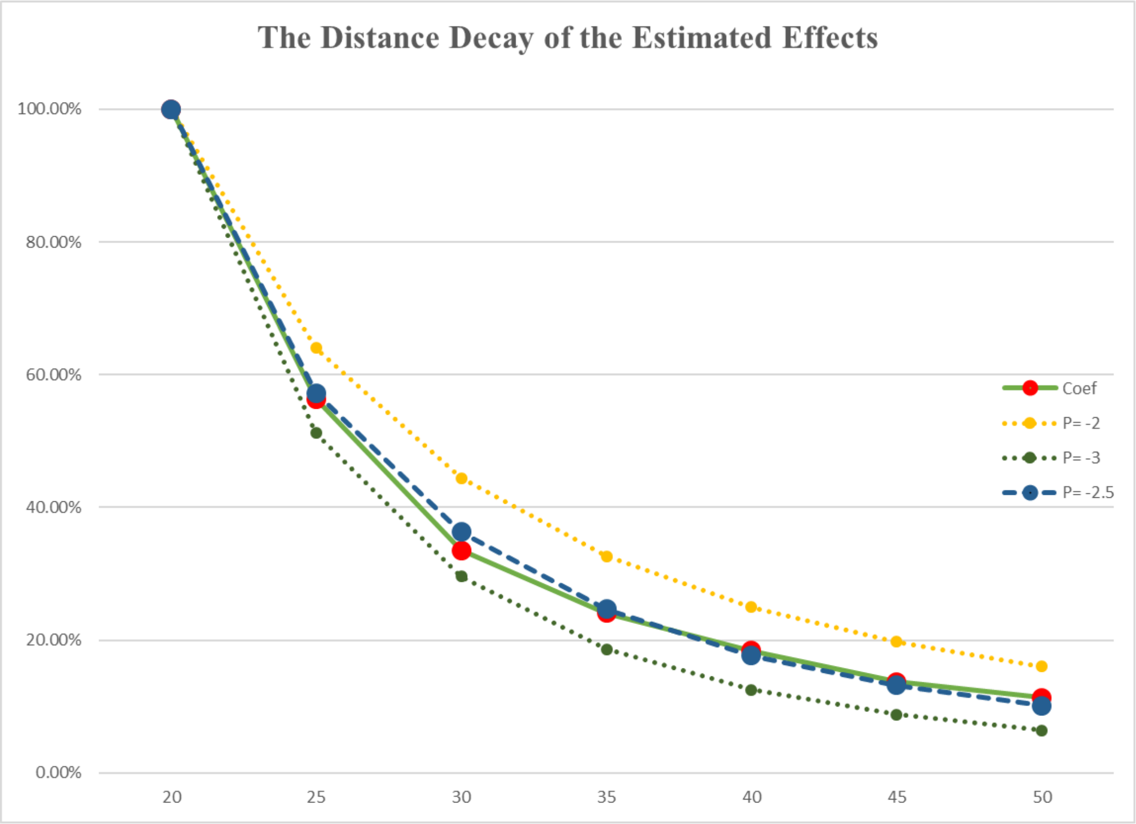

Supplementary Figure 5. The observed distance-dependent decay of the estimated effects and the modeled distance decay by power functions with negative exponents.

Y-axis represents the ratio between the estimated effects at the specific distance and the estimated effects at 20 km. The solid curve indicates the observed distance-decay of the estimated effects (“coef”). We observed an apparent distance-dependent decay in the effects of UOGD on PR (**Figure 2**). This trend suggests that we could tentatively extrapolate our results to a finer spatial scale. We used a power function with a negative exponent to fit the decay. The exponent with the best fit is -2.5, between -2 (indicates a two-dimensional dispersion) and -3 (indicates a three-dimensional dispersion).

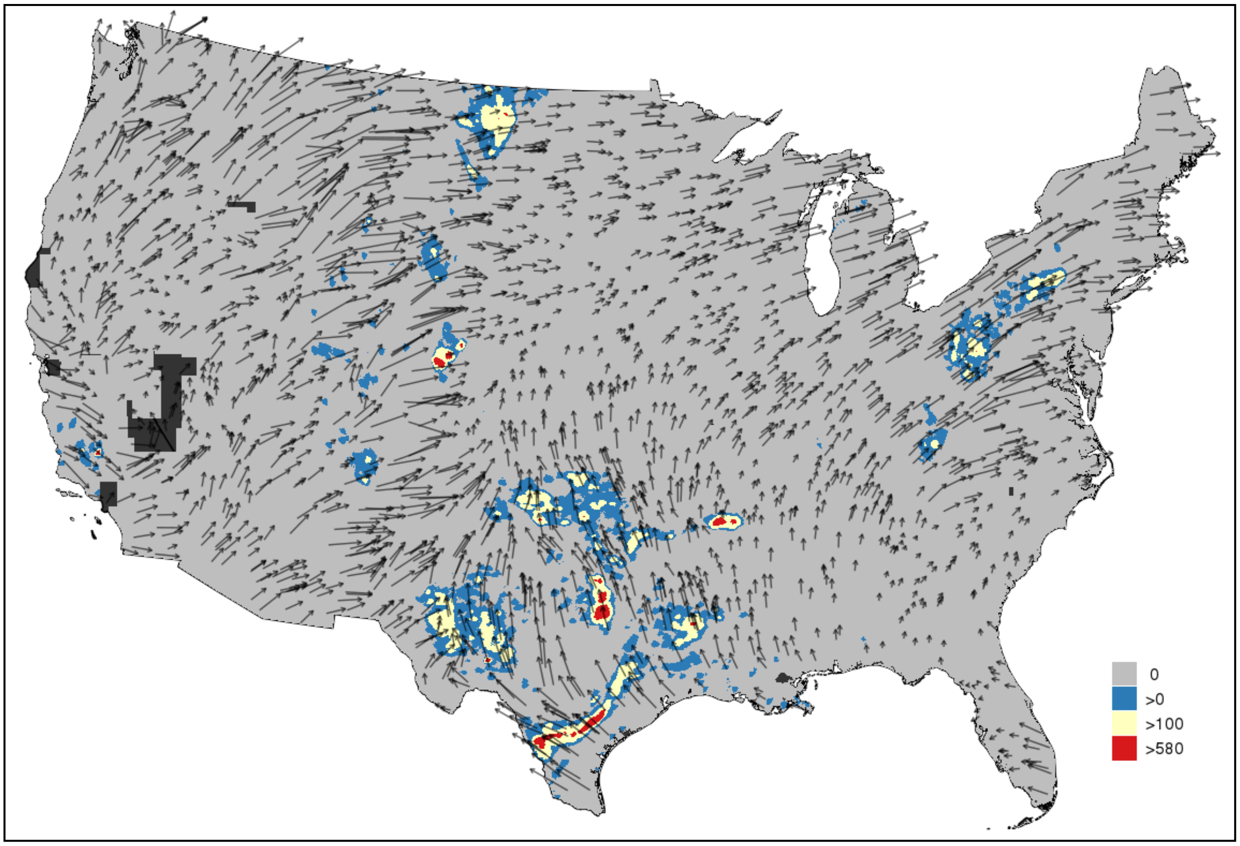

Supplementary Figure 6. Regions with annual average upwind UOGD wells over 580.

Wind vectors (black arrows in the figure) indicate the annual prevailing wind field, which was used to calculate upwind O&G development. UOGD wells used to calculate upwind activities were completed before 12/31/2017.

As shown in **Figure 1**, the majority of UOGD wells are not drilled close to metropolitan regions. Likely, the PR levels in some communities distant from RadNet monitors could also be elevated by the extensive UOGD activities nearby. To identify these communities, we mapped the 1 km grids whose annually-averaged upwind UOGD well count was over 580 (the 95% percentile of upwind UOGD well count from the modelling area) and over 100 at the end of 2017. There are several regions, including the core part of Eagle Ford shale, Barnett shale, Fayetteville shale, and Niobrara shale, with an annual average UOGD over 580. The total area of these regions is 10,724 km^2. Over 1.2 million people reside in these areas. Based on the health studies cited in the main text, residents living in these communities are more likely to show adverse symptoms, including higher blood pressure, decreased lung function, and increased level of the inflammatory biomarker, if the PR increase downwind of UOGD wells in these regions are consistent with those of our study.

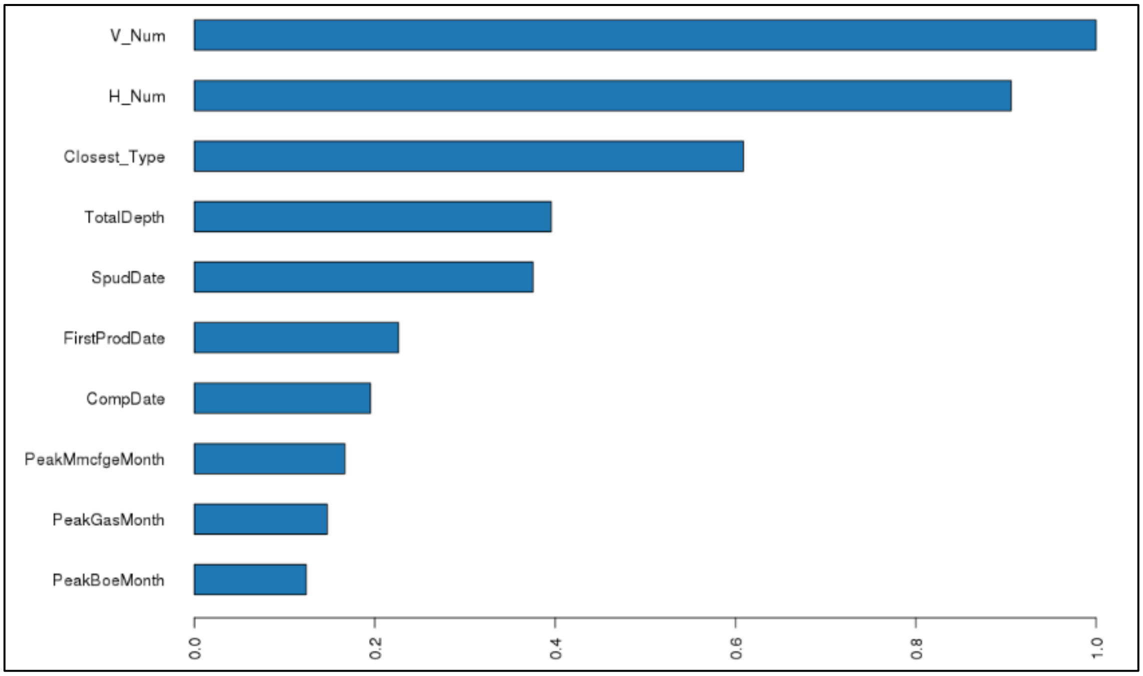

Supplementary Figure 7. Relative importance of covariates in the random forest model.

Full names of the variables from top to bottom: Number of conventional wells within 10km, number of unconventional wells within 10 km, the drilling type of the closest well with known drilling type, total drilling depth, spudding date, the date of the first production record, completion date, the date of peak million cubic feet of gas equivalent production, the date of peak natural gas production, the date of peak barrel of oil equivalent production.

Supplementary Tables

Supplementary Table 1. Annual average upwind UOGD count in three representative cities of three subregions.

	Pittsburg, PA		Fort Worth, TX		Navajo Lake, NM	
	20 km	50 km	20km	50km	20km	50km
2000	0.0	0.0	0.0	0.0	0.0	0.0
2001	0.0	0.0	0.0	0.0	0.0	0.0
2002	0.0	0.0	0.0	0.0	0.0	0.0
2003	0.0	0.0	0.0	0.0	0.0	0.0
2004	0.0	0.0	0.0	0.0	0.0	0.0
2005	0.0	0.0	0.0	0.0	0.0	0.0
2006	0.0	2.5	0.0	0.0	0.0	0.0
2007	0.0	5.7	143.1	980.2	0.0	0.0
2008	0.0	24.0	216.6	1561.0	0.0	0.0
2009	0.0	41.2	293.6	2020.6	0.0	0.0
2010	0.0	105.5	372.4	2502.8	43.0	111.5
2011	1.0	174.1	466.3	3076.5	46.9	112.6
2012	1.0	232.0	523.6	3179.9	47.6	135.0
2013	1.9	314.3	533.4	3060.2	53.8	155.8
2014	11.8	380.8	559.0	3143.0	45.0	141.3
2015	12.0	456.0	572.0	3196.5	55.1	154.9
2016	12.0	540.2	573.1	3095.0	56.9	155.7
2017	12.0	575.1	585.8	3240.9	59.3	162.8

Most O&G wells, especially UOGD wells, are relatively distant from metropolitan areas where the RadNet monitors are located. As a result, the average upwind UOGD well count does not fully represent the temporal trend of UOGD expansion. We selected one RadNet monitor from each of the three subregions to represent the temporal trend of upwind UOGD well count.

Supplementary Table 2. Source data for Figure 2 in the main text

Variable	Radius (km)	Coefficient (mBq/m^3 per 100 wells)	95% CI Lower Bound	95% CI Upper Bound
Upwind UOGD	20	2.40E-02	1.97E-02	2.82E-02
Upwind UOGD	25	1.35E-02	1.11E-02	1.59E-02
Upwind UOGD	30	8.05E-03	6.54E-03	9.55E-03
Upwind UOGD	35	5.78E-03	4.69E-03	6.87E-03
Upwind UOGD	40	4.43E-03	3.57E-03	5.28E-03
Upwind UOGD	45	3.29E-03	2.62E-03	3.96E-03
Upwind UOGD	50	2.72E-03	2.15E-03	3.28E-03
Upwind COGD	20	3.60E-03	2.91E-03	4.29E-03
Upwind COGD	25	5.27E-04	-9.41E-06	1.06E-03
Upwind COGD	30	-2.39E-04	-5.49E-04	7.02E-05
Upwind COGD	35	-7.67E-05	-2.64E-04	1.10E-04
Upwind COGD	40	9.85E-08	-1.52E-04	1.53E-04
Upwind COGD	45	7.63E-05	-5.22E-05	2.05E-04
Upwind COGD	50	8.15E-05	-2.77E-05	1.91E-04

Supplementary Table 3 The results of analysis to estimate the temporal variation in the influence of UOGD

**Sub-period**	**Coefficient (mBq/m^3 per 100 wells)**	**95% CI Lower Bound**	**95% CI Upper Bound**
2001~2011	5.15E-02	4.46E-02	5.84E-02
2003~2014	1.96E-02	1.54E-02	2.38E-02
2006~2017	1.68E-02	1.35E-02	2.02E-02
2008~2017	1.19E-02	8.21E-03	1.55E-02

We refitted the primary model for four sections of our study period: from 2001 to 2014, from 2001 to 2011, from 2006 to 2017, and from 2008 to 2017. It seems that the influence of per 100 UOGD wells decreased gradually in our study period. However, this result should be interpreted with caution because it is difficult to disentangle the temporal trend and spatial pattern of UOGD.

Supplementary Table 4. The source data table of Supplementary Figure 2 and the results of negative control analysis

**Wind Direction**	**Central Angle of the buffer (Degree)**	**Radius (km)**	**Coefficient (mBq/m^3 per 100 wells)**	**95% CI Lower Bound**	**95% CI Upper Bound**
Upwind	30	20	3.38E-02	2.76E-02	4.01E-02
Upwind	45	20	2.40E-02	1.97E-02	2.82E-02
Upwind	60	20	1.80E-02	1.49E-02	2.12E-02
Upwind	45	25	1.35E-02	1.11E-02	1.59E-02
Upwind	30	25	1.88E-02	1.54E-02	2.22E-02
Upwind	60	25	1.08E-02	8.97E-03	1.27E-02
Upwind	45	30	8.05E-03	6.54E-03	9.55E-03
Upwind	30	30	1.03E-02	8.27E-03	1.24E-02
Upwind	60	30	7.01E-03	5.80E-03	8.23E-03
Upwind	45	35	5.78E-03	4.69E-03	6.87E-03
Upwind	30	35	7.20E-03	5.74E-03	8.66E-03
Upwind	60	35	5.19E-03	4.29E-03	6.09E-03
Upwind	45	40	4.43E-03	3.57E-03	5.28E-03
Upwind	30	40	5.46E-03	4.32E-03	6.61E-03
Upwind	60	40	4.04E-03	3.32E-03	4.76E-03
Upwind	45	45	3.29E-03	2.62E-03	3.96E-03
Upwind	30	45	4.10E-03	3.20E-03	4.99E-03
Upwind	60	45	3.00E-03	2.44E-03	3.57E-03
Upwind	45	50	2.72E-03	2.15E-03	3.28E-03
Upwind	30	50	3.38E-03	2.62E-03	4.13E-03
Upwind	60	50	2.46E-03	1.99E-03	2.94E-03

Supplementary Table 4 (Continued) The source data table of Supplementary Figure 2 and the results of negative control analysis

**Wind Direction**	**Central Angle of the buffer (Degree)**	**Radius (km)**	**Coefficient (mBq/m^3 per 100 wells)**	**95% CI Lower Bound**	**95% CI Upper Bound**
Downwind	30	20	2.77E-02	2.23E-02	3.32E-02
Downwind	45	20	2.05E-02	1.68E-02	2.42E-02
Downwind	60	20	1.74E-02	1.46E-02	2.02E-02
Downwind	45	25	1.74E-02	1.40E-02	2.09E-02
Downwind	30	25	1.33E-02	1.10E-02	1.57E-02
Downwind	60	25	1.10E-02	9.20E-03	1.28E-02
Downwind	45	30	1.16E-02	9.49E-03	1.38E-02
Downwind	30	30	9.49E-03	7.92E-03	1.11E-02
Downwind	60	30	7.98E-03	6.75E-03	9.21E-03
Downwind	45	35	9.68E-03	8.13E-03	1.12E-02
Downwind	30	35	8.07E-03	6.93E-03	9.22E-03
Downwind	60	35	6.94E-03	6.01E-03	7.86E-03
Downwind	45	40	7.88E-03	6.68E-03	9.08E-03
Downwind	30	40	6.53E-03	5.64E-03	7.42E-03
Downwind	60	40	5.65E-03	4.93E-03	6.38E-03
Downwind	45	45	5.71E-03	4.79E-03	6.62E-03
Downwind	30	45	4.70E-03	4.02E-03	5.37E-03
Downwind	60	45	4.10E-03	3.55E-03	4.65E-03
Downwind	45	50	4.46E-03	3.71E-03	5.20E-03
Downwind	30	50	3.67E-03	3.12E-03	4.22E-03
Downwind	60	50	3.23E-03	2.77E-03	3.68E-03
